# Pre-operative Halo-Pelvic Traction for Neurofibromatosis Patients with Severe Proximal Thoracic Spinal Deformity: Indications and Early Treatment Outcome

**DOI:** 10.5704/MOJ.2111.015

**Published:** 2021-11

**Authors:** WH Chung, Y Mihara, SS Toyat, CK Chiu, MS Hasan, A Saw, CYW Chan, MK Kwan

**Affiliations:** 1 Department of Orthopaedic Surgery (NOCERAL), University of Malaya, Kuala Lumpur, Malaysia; 2 Department of Orthopaedic Surgery, Hamamatsu University School of Medicine, Hamamatsu-shi, Japan; 3 Department of Anaesthesiology, University of Malaya, Kuala Lumpur, Malaysia

**Keywords:** halo-pelvic, traction, neurofibromatosis, proximal thoracic, deformity

## Abstract

**Introduction:**

To report the indications and early treatment outcomes of pre-operative halo-pelvic traction in patients with neurofibromatosis associated with severe proximal thoracic (PT) spinal deformity.

**Materials and methods:**

We reviewed four patients with neurofibromatosis with severe PT spinal deformity. Case 1, a 16-year-old male presented with severe PT kyphoscoliosis (scoliosis: 89°, kyphosis: 124°) and thoracic myelopathy. Case 2 was a 14-year-old, skeletally immature male who presented with a PT lordoscoliosis (scoliosis: 85°). Case 3, a 13-year-old male, presented with severe PT kyphoscoliosis (scoliosis: 100°, kyphosis: 95°). Case 4, a 35-year-old gentleman, presented with severe PT kyphoscoliosis (scoliosis: 113°, kyphosis: 103°) and thoracic myelopathy. All patients underwent pre-operative halo-pelvic traction. After a period of traction, all patients underwent posterior spinal fusion (PSF) with autologous bone grafts (local and fibula bone grafts) and recombinant human bone morphogenetic protein-2 (rhBMP-2).

**Results:**

Both patients with thoracic myelopathy regained near normal neurological status after halo-pelvic traction. Following traction, the scoliosis correction rate (CR) ranged from 18.0% to 38.9%, while the kyphosis CR ranged from 14.6% to 37.1%. Following PSF, the scoliosis CR ranged from 24.0% to 58.8%, while the kyphosis CR ranged from 29.1% to 47.4%. The total distraction ranged from 50-70mm. Duration of distraction ranged from 26-95 days. The most common complication encountered during halo-pelvic traction was pin-related e.g. pin tract infection, pin loosening and migration, osteomyelitis, and halo-pelvic strut breakage. No patients had cranial nerve palsies or neurological worsening.

**Conclusion:**

Pre-operative correction of severe PT spinal deformities could be performed safely and effectively with the halo-pelvic device prior to definitive surgery.

## Introduction

The incidence of spinal deformity in neurofibromatosis is reported to be between 10% to 60%^1^. The spinal deformity can be either non-dystrophic or dystrophic. Dystrophic deformity is characterised by a short-segmented (4-6 vertebrae), sharp angular curve most frequently associated with kyphosis and a higher incidence of neurologic injury^2^.

Dystrophic neurofibromatosis is resistant to bracing^3^ and has a tendency for rapid curve progression^4^. Therefore, early and aggressive surgical intervention is usually required to halt further progression of the deformity^5^. However, correction of the dystrophic spinal deformity in neurofibromatosis patients is not only difficult, but carries much higher risks of neurologic complications, bleeding, pseudoarthrosis, and implant failure necessitating a revision surgery^6-9^.

Pre-operative halo-gravity traction is commonly used for patients with severe spinal deformity. However, the halo-gravity technique is less effective in improving the curve flexibility in severe and rigid spinal deformity. Halo-pelvic traction is a form of pre-operative traction method which had been described in the past but is less commonly utilised nowadays^10^. In this case series, we would like to revisit the use of this old technique in four patients with difficult neurofibromatosis who presented with severe proximal thoracic (PT) spinal deformity with and without neurological deficit.

## Materials and Methods

This is a case series to report four patients with neurofibromatosis with severe PT spinal deformity who had undergone a period of gradual deformity correction using the halo-pelvic device prior to definitive surgery between 2017 and 2019. The halo-pelvic device was applied to allow preoperative gradual correction of the spinal deformity to reduce the risk of implant failure as well as to minimise the risk of intra-operative neurologic events. The halo-pelvic device was also useful to delay the definitive surgery in skeletally immature patients. The demographic data and perioperative data such as age, gender, Risser grade, height, weight, body mass index, Cobb angle of the major curve (before traction, after traction and after surgery), kyphosis (before traction, after traction and after surgery), correction rate, number of fusion levels, operation time and estimated blood loss are included. Parameters regarding the halo-pelvic traction such as number of halo and pelvic pins, patient’s weight before and after traction, weight gained, total distraction, duration of the distraction, distraction per day and duration of patients on halo-pelvic frame were reviewed. Patients consented to participate in this case series.

### Case 1

**Fig 1:**
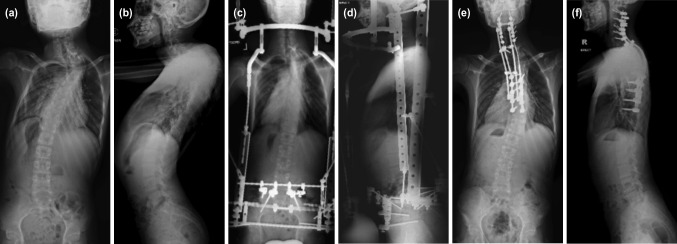
Plain radiographs (posteroanterior or and lateral view) of Case 1: (a, b) before operation, (c, d) while on halo-pelvic traction, and (e, f) after single-stage posterior spinal fusion.

**Fig 2:**
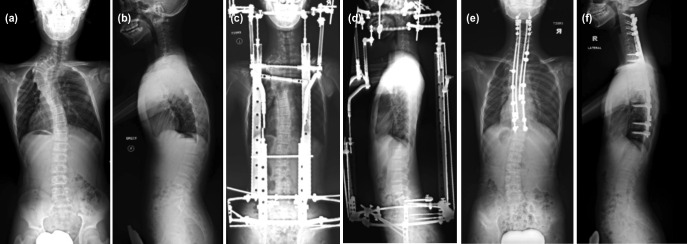
Plain radiographs (posteroanterior and lateral view) of Case 2: (a, b) before operation, (c, d) while on halo-pelvic traction, and (e, f) after single-stage posterior spinal fusion.

This patient was a 16-year-old male, with neurofibromatosis type 1, who presented with severe PT kyphoscoliosis. He presented with thoracic myelopathy with sensory level at T5 and Medical Research Council (MRC) grade 4 power in both lower limbs. Plain radiographs of the whole spine showed a scoliosis of 89° and kyphosis of 124° (Fig. 1a and b). Halo-gravity traction was applied initially, however, the treatment failed due to poor compliance. Later, a halo-pelvic traction device was applied. He regained near normal neurological function after six months of distraction and immobilisation with the halo-pelvic frame (Fig. 1c and d). He underwent insitu single-staged PSF (using 4-rod technique) from C2 to T10 level (Fig. 1e and f). Autologous local bone graft, and autologous fibula bone graft and rhBMP-2 (Medtronic INFUSE bone graft) were used to augment the fusion process. A second-staged anterior spinal fusion using a fibula strut graft was planned. However, the pre-operative computed tomography (CT) angiogram showed that the left brachiocephalic vein had an anomalous retro-oesophageal and retro-tracheal course. The vein was anterior to the apex of the PT kyphosis where the anchor site for the fibula graft was planned (Fig. 2). Therefore, the second stage procedure was abandoned.

### Case 2

This was a 14-year-old male, with neurofibromatosis type 1, who presented with a PT lordoscoliosis. Whole spine plain radiographs showed a Cobb angle of 85° (Fig. 2a and b). At the time of presentation, he was immature with the triradiate cartilages still open bilaterally. A decision was made to apply the halo-pelvic frame to delay definitive surgery until the closure of the triradiate cartilages (Fig. 2c and d). The definitive surgery was delayed for eight months. A single-staged PSF (using 4-rod technique) from C2-T12 was performed (Fig. 2e and f). Autologous bone grafts (local and fibula grafts) and rhBMP-2 were used to augment the fusion process.

### Case 3

**Fig 3:**
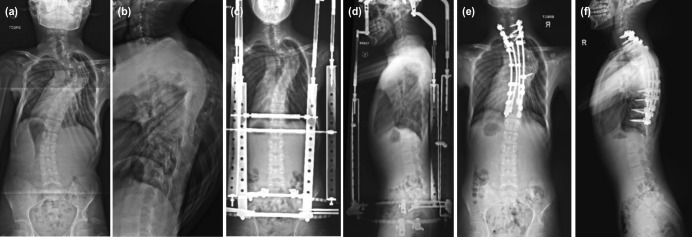
Plain radiographs (posteroanterior and lateral view) of Case 3: (a, b) before operation, (c, d) while on halo-pelvic traction, and (e, f) after single-stage posterior spinal fusion.

The patient was a 13-year-old male who presented with a severe PT kyphoscoliosis without neurological deficit. Plain radiographs of the whole spine showed severe scoliosis measuring 100° and kyphosis of 95° (Fig. 3a and b). Halo-pelvic traction was applied. Post-distraction, his scoliosis was 82° with kyphosis of 73° (Fig. 3c and d). He underwent single-staged PSF (using 4-rod technique) from C7-T12, augmented with autologous bone grafts (local and fibula grafts) and rhBMP-2 (Fig. 3e and f).

### Case 4

**Fig 4:**
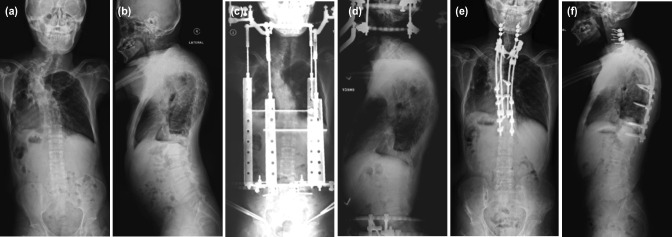
Plain radiographs (posteroanterior and lateral view) of Case 4: (a, b) before operation, (c, d) while on halo-pelvic traction, and (e, f) after single-stage posterior spinal fusion.

**Fig 5:**
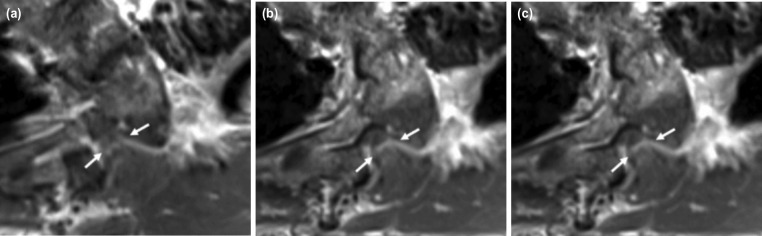
(a, b, c) MRI of the spine of Case 4 showing cord compression at T4/5 level due to a neurofibroma (white arrow).

**Fig 6:**
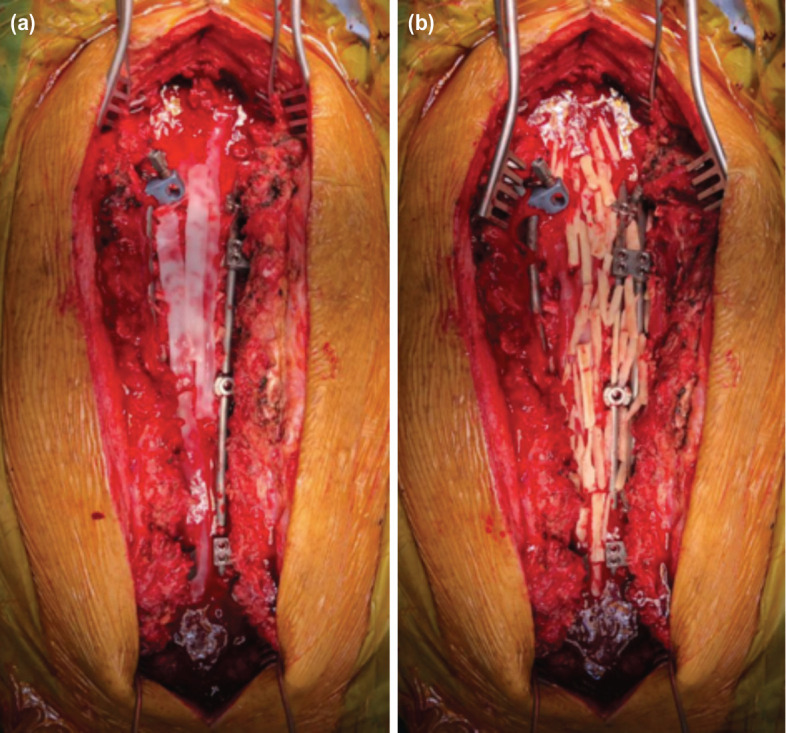
(a) Intra-operative photographs showing the rh-BMP-2 strips were applied over the layer of onlay autologous local bone graft (b) and strips of autologous fibula bone graft were applied on top of the rh-BMP-2 strips.

The patient was a 35-year-old male who presented with a severe PT kyphoscoliosis with worsening thoracic myelopathy and urinary incontinence i.e. Frankel D neurology following a fall. Physical examination revealed reduced sensation below T5 level and motor power (MRC grade 4) in both lower limbs. Whole spine plain radiographs showed scoliosis measuring 113° with kyphosis of 103° (Fig. 4a and b). Magnetic resonance imaging (MRI) showed concomitant cord compression at the apex of deformity (T4/T5 level) secondary to a neurofibroma at that level (Fig. 5). Halo-gravity traction was applied. However, his lower limb numbness worsened whenever the traction weight was increased. We postulated that the neurological worsening was contributed by mobility over the apex of the kyphoscoliosis coupled with spinal cord compression by the neurofibroma. A halo-pelvic traction was applied to provide spinal stability with gradual correction of the deformity (Fig. 4c and d). After one month of distraction, his neurological status improved (improvement of sensation and power in both lower limbs recovered to MRC grade 5). A single-staged PSF (using 4-rod technique) from C3-T12 level and excision of the neurofibroma was performed (Fig. 4e and f), with augmentation with autologous bone grafts (local and fibula grafts) and rhBMP-2 similar to previous cases (Fig. 6).

While the patients were in the hospital, the primary focus of the patients’ management was divided into: (i) pain management; (ii) distraction protocol; (iii) rehabilitation programme; and lastly (iv) detection of complications and management. Patients were given either oral (preferably) or intravenous analgesics for the first few weeks and later whenever needed. The distraction regime started with daily distraction of 2mm for the first 1 to 2 weeks. Following that, the distraction was performed once every two to three days depending on patient’s tolerance. As for the rehabilitation programme, patients were encouraged to ambulate with guidance from physiotherapists, in addition to limb strengthening exercises and balancing exercises. Daily assessment of complications such as neurological deficits, cranial nerve dysfunction, neck pain and pin-related complications were carried out and each complication was managed accordingly.

## Results

Table I shows the patient demographics, radiological data as well as intra-operative parameters for the patients. Three out of four patients presented with kyphoscoliotic deformity. The mean correction rate of scoliosis following traction and definitive surgery was 32.6% (range: 18 to 38.9%) and 41.5% (range: 24 to 58.8%), respectively. For kyphosis, the mean correction rate following traction and definitive surgery was 24.5% (range: 14.6 to 37.1%) and 36.9% (range: 29.1 to 47.4%), respectively.

**Table I:** Demographic and perioperative data

Parameters	Mean (range)	Case 1	Case 2	Case 3	Case 4
Age (years)	19.5 (13-35)	16	14	13	35
Gender	-	Male	Male	Male	Male
Risser grade	-	4	0	3	5
Height (cm)	155.5 (153.1-157)	153.1	156	156	157
Weight (kg)	44 (40.4-52.8)	41.4	40.4	41.4	52.8
BMI (kg/m2)	18.2 (16.6-21.4)	17.7	16.6	17	21.4
Pre traction Cobb angle (°)	96.8 (85-113)	89	85	100	113
Post traction Cobb angle (°)	65 (52-82)	58	52	82	69
Post traction Cobb CR (%)	32.6 (18-38.9)	34.8	38.8	18	38.9
Post-operative Cobb angle (°)	57 (35-76)	55	35	76	62
Post-operative Cobb CR (%)	41.5 (24-58.8)	38.2	58.8	24	45.1
Pre traction Kyphosis (°)	83.8 (13-124)	124	13	95	103
Post traction Kyphosis (°)	62 (10-88)	78	10	73	88
Post traction Kyphosis CR (%)	24.5 (14.6-37.1)	37.1	23.1	23.2	14.6
Post-operative Kyphosis (°)	51.5 (9-74)	74	9	50	73
Post-operative Kyphosis CR (%)	36.9 (29.1-47.4)	40.3	30.8	47.4	29.1
Number of fusion levels	15 (12-17)	15	17	12	16
Operation time (minutes)	311.3 (250-400)	400	255	250	340
EBL (ml)	1534 (1100-1800)	1740	1496	1100	1800

Abbreviations – BMI: body mass index, Pre traction: pre halo-pelvic traction, Cobb angle: Cobb angle of the major curve, CR: correction rate, EBL: estimated blood loss

For Case 2, the scoliosis improved by 38.8% using the halo-pelvic device and following surgery, the correction rate was 58.8%. The post-operative correction rate for scoliosis in Case 1, 3 and 4 ranged from 24.0% to 45.1%. In Case 1, the pre-operative kyphosis was the most severe measuring 124°. Using the halo-pelvic device, the kyphosis was corrected to 78° with a correction rate of 37.1%. This was in comparison to Case 3 and 4 whereby only a modest correction of the kyphosis was obtained following halo-pelvic traction at 23.2% and 14.6%, respectively. Nevertheless, following surgery, we obtained a kyphosis correction rate of 47.4% and 29.1% in Case 3 and 4, respectively. The operative time for all four cases ranged from 250 to 400 minutes with an estimated blood loss ranging from 1100 to 1800mls.

The total amount of distraction length ranged from 50mm to 70mm with an average duration of 64 days (approximately 2 months), and ranged from 26 to 95 days. The distraction regime started with daily distraction of 2mm for the first 1 to 2 weeks. Following that, the distraction was performed once every 2 to 3 days depending on the patient’s tolerance. The average distraction for Case 1, 3 and 4 was 0.8mm per day. For Case 2, the distraction rate was higher because the halo-pelvic system was used as a growing device as the patient was still immature (Risser 0). The average total duration the patients were on the halo-pelvic frame was 211 days, approximately 7 months (Table II).

**Table II:** Halo-Pelvic Traction Parameters

Parameters	Case 1	Case 2	Case 3	Case 4
Number of halo pins	8	8	8	4
Number of pelvic pins	8	6	6	8
Weight before traction* (kg)	43.0	39.3	39.5	55
Weight after traction* (kg)	46.0	40.4	41.4	60
Weight gained (kg)	3	1.1	1.9	5
Total distraction (mm)	65	70	50	65
Duration of distraction (days)	78	26	57	95
Distraction per day (mm/day)	0.8	2.7	0.9	0.7
Duration on halo-pelvic frame (days)	330	220	72	223

*Patient’s weight including the weight of the halo-pelvic frame

**Fig 7:**
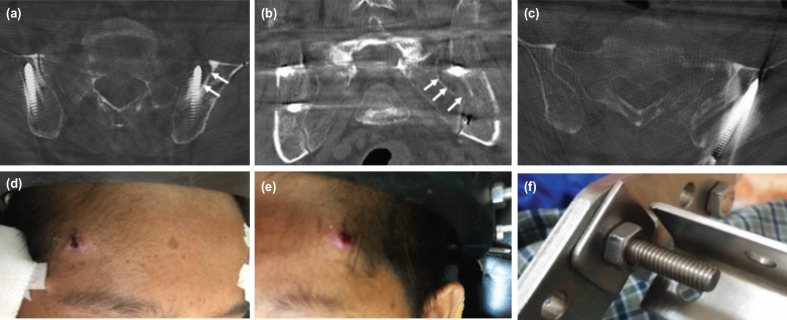
(a, b c) CT pelvis of Case 4 showing septic loosening with osteomyelitis (white arrow). (d, e) Photographs showing loosening and migration of anterior halo pins in Case 2. (f) Showed a broken halo-pelvic strut.

All the patients had more than one complication during the treatment using halo-pelvic traction. The most common complication encountered was pin-related. All the cases experienced some forms of pin tract infection, which fortunately could be easily treated with daily dressing and intravenous or oral antibiotics. In addition, one of the pelvic pins in Case 1 loosened and was subsequently removed. In Case 4, the patient had to undergo pelvic pin revision twice due to septic loosening with osteomyelitic changes, which were noted in his CT scan images (Fig. 7a, b and c). In Case 2, the patient experienced loosening and migration of the anterior halo pins which required reapplication (Fig. 7d and e). Halo pelvic strut breakage was noted in Case 2 (Fig. 7F).

## Discussion

The optimal surgical management for severe dystrophic spinal deformity in neurofibromatosis is debatable. Posterior surgery alone was reported to be associated with higher risk of pseudoarthrosis^6,7,8^. A combined anterior/posterior approach was recommended to reduce the risk of non-union^9,11^. However, acute correction with combined anterior/posterior surgeries or posterior only approaches utilising posterior column osteotomies were often associated with higher perioperative morbidities. Excessive intra-operative bleeding can occur in anterior approaches because of the presence of plexiform venous plexus in the paraspinal soft tissues^7,9^. Anterior surgery may also result in pulmonary compromise including lung collapse, pneumonia and hemothorax^9^. Due to the morbidities associated with anterior approaches, posterior vertebral column resection had been proposed^12,13^. This was a technically demanding procedure with high risk of neurologic and non-neurologic complications even in specialised centres^13,14^. In a large multicentre series of 147 patients, the risk of intra-operative neurologic events was 27%^14^. Yang *et al* also reported the risk of neurologic complications was 8% with 2% risk of acute spinal cord injury^15^.

Previous authors who employed combined anterior-posterior approach for severe spinal deformities reported various complications. Bullmann *et al* reported post-operative pleural effusion in 33 patients^16^. Shen *et al* compared single-staged vs. two-staged surgery and documented one episode of pneumonia, one of pneumothorax and one of ileus in the perioperative period^17^. Kandwal *et al* reported a complication rate of 33.3% including in two patients who had basal atelectasis and prolonged need for a chest drain^18^. In a multicentre study of 147 patients who underwent vertebral column resection (VCR), Lenke *et al* highlighted the complication rate as high as 59%, with 27% rate of intra-operative neurological events^14^. Riley *et al* assessed 109 patients who underwent posterior VCR for severe spinal deformities. Out of 54 patients who were available for analysis, Riley *et al* reported 55.6% of patients had postoperative complications and 9.3% developed post-operative neurological deficits^13^. Suk *et al* performed single-staged VCR in 16 patients with severe, rigid scoliosis, and documented one case of acute complete paralysis, one case of hematoma and one of haemopneumothorax^19^.

An alternative surgical strategy that could be employed to optimise patients prior to definitive surgery is halo-gravity traction. Although this technique was effective in improving patients’ pulmonary function and nutritional status, it was not as effective when used as a tool to increase the flexibility of a rigid curve. In a review of 21 patients who underwent halo-gravity traction, the correction of kyphosis before definitive surgery was a modest 25%^20^. Similarly, Koller *et al* noted that the mean flexibility of severe scoliosis curves during halo-gravity traction was an average 14.8%, similar to the flexibility assessment using bending or traction radiographs^21^. Perry and Nickel first described the use of the halo skeletal fixator in the 1950s for postsurgical stabilisation of patients with poliomyelitis who underwent cervical fusion surgery^22,23^. The halo-tibial and halo-femoral traction was later introduced to provide more sustained and powerful counter traction^24^. Dewald and Ray, later designed the “University of Illinois halo hoop apparatus” which included a hoop that attached the pelvis with iliac rods and the hoop was then attached to the halo with turnbuckles^25^. It was later popularised as the halo-pelvic device and was used in over 100 patients with severe tuberculous kyphotic and paralytic scoliotic patients^26^.

There has not been many recent reports on the use of halo-pelvic device prior to definitive surgery. The halo-pelvic device is a powerful tool for pre-operative correction of the kyphotic deformity. This would allow less intra-operative manipulation of the spine. This was advantageous as it would apply less biomechanical stress on the construct and also will reduce the risk of neurologic events during the surgery. In patients who presented with thoracic myelopathy, Case 1 and 4 illustrated the advantages of using halo-pelvic device compared to halo-gravity traction. A halo-pelvic device provided spinal stability once applied. This was an important factor to prevent worsening of the neurological status when traction was applied as demonstrated in Case 4. Furthermore, powerful correction of the kyphotic deformity also leads to less compression to the spinal cord by the internal kyphus. In both patients, they regained near normal neurological status prior to the definitive surgery. Another scenario whereby this device could be useful was illustrated in Case 2, the patient was skeletally immature and definitive fusion could not be performed. The halo-pelvic device was then used to delay the definitive surgery. At the time of definitive surgery, the patient’s triradiate cartilage was closed. However, he seemed to be still skeletally immature and therefore, he was at a higher risk of crankshaft phenomenon as described in previous reports^27,28^.

The halo-pelvic traction acts as a useful tool for preoperative kyphotic correction prior to definitive surgery especially in (i) patients who are skeletally immature; (ii) patients with pre-existing neurological deficit or at higher risk of neurological deficit during corrective surgery; and (iii) severe and rigid coronal and/ or sagittal deformity.

One important technical note we would like to highlight about the distraction regime of the halo-pelvic device is that the distraction should be started at a faster pace with daily distraction of 2mm for the first 1 to 2 weeks. After which, the distraction should be slowed down and be performed once every two to three days depending on the patient’s pain tolerance level. However, in patients who are skeletally immature, the halo-pelvic device can be used as a growing tool, and therefore, the distraction rate can be higher.

Neurofibromatosis patients with dystrophic scoliosis are predisposed to rib head protrusion into the spinal canal^29,30^. The intraspinal rib head protrusion may cause spinal cord compression leading to neurological deficits both before and after corrective surgeries^29^. However, there is no consensus regarding whether to resect the protruded rib head during the corrective surgeries^30^. In our case series, 50% of the patients had rib head protrusion. Case 1 had rib head protrusion to T4 vertebra at the convex side. Case 3 had rib head protrusion to T5 and T6 vertebrae at the convex side as well. However, there was no cord compression by the intraspinal rib heads. Ton *et al* described that corrective surgeries could bring the displaced spinal cord to its more anatomical location and result in spinal cord compression by the unrecognised protruded rib head^29^. Both cases in this series illustrate that the halo-pelvic traction allows gradual distraction in this group of patients without risking spinal cord compression by the intraspinal rib heads.

The distraction process in our cases was most commonly complicated by pin-related issues. This was consistent with Ransford *et al* who reported their experience in 118 scoliosis patients who were placed in halo-pelvic devices. In their series, the pelvic pins commonly caused more problems, particularly the anterior pins^31^. We also encountered similar issues as reported above. All cases had pin tract infection. The most severe case of pin tract infection occurred in case 4 whereby the pins were loosened and there were osteomyelitic changes in the bone. In case 2, the distraction forces caused the halo pin migration and dislodgement. The same patient also had one of the struts broken that was replaced. No patients experienced any cranial nerve lesions or neurological worsening. In our experience, pin tract infection can be managed with dressing and/ or antibiotics. However, loosened pins should be removed. The halo-pelvic distraction should be withheld until new pins are inserted at the new sites. The versatility of the halo and pelvic rings with multiple holes allows new pins to be inserted at new sites without much adjustment of the frames.

## Conclusion

This is a report of four patients with neurofibromatosis with severe proximal thoracic spinal deformities who underwent gradual pre-operative halo-pelvic traction prior to a definitive posterior spinal fusion (4-rod technique) surgery. Pre-operative correction of severe proximal thoracic spinal deformities could be performed safely and effectively with the halo-pelvic device prior to definitive surgery.
